# The impact of perceived human and computer interaction on collaborative learning: insights from the Map Task

**DOI:** 10.1007/s10339-026-01347-3

**Published:** 2026-05-27

**Authors:** Catherine J. Crompton, Kelly Wolfe, Alisdair Tullo, Paul Hoffman, Maria K. Wolters, Sarah E. MacPherson

**Affiliations:** 1 Human Cognitive Neuroscience, Department of Psychology, University of Edinburgh, 7 George Square, Edinburgh, EH8 9JZ UK; 2 Division of Psychiatry, Centre for Clinical Brain Sciences, University of Edinburgh, Edinburgh, UK; 3 School of Informatics, University of Edinburgh, Edinburgh, UK

**Keywords:** learning, aging, human-computer interaction, collaboration, human-centered computing, empirical studies in accessibility, older adults, recall

## Abstract

Learning new skills and information becomes more challenging as people get older, but learning collaboratively can provide benefits to learning in older age. Prior work has predominantly focused on improved learning in terms of accuracy and efficiency when interacting with another human compared to learning alone, while the potential benefits of a human learning with a computer are understudied. It has been reported that older adults learn more efficiently and accurately when they believe they are interacting with a human compared to a computer to perform the Barrier Task. In the current study, we examined the effect of agency on performance on the Map Task, a test that measures spatial cognition, where participants must orally collaborate to reproduce a route printed on one participant’s map onto another’s, and which is considered a more ecologically valid measure of real-life communication compared to other materials used to study collaborative learning. We used a Wizard-of-Oz paradigm, in which participants interacted with a computer system, but were told that they were interacting with either a human assistant or computer. Twenty-four older adult participants (aged 66–79 years) completed an adapted version of the Map Task with a (perceived) human partner or computer to plan and execute a route, doing so through conversation. Overall, participants engaged similarly with their human and computer partner, but recall was more accurate when the information had been provided by the human, compared to the computer. Participants’ negative perceptions about the computer-provided information may have driven this effect. These findings may influence how voice-based learning systems are introduced to older end-users, and strategies for negotiating agency between the human user and the voice-based system, to influence collaborative learning in older age. Future work should attempt to replicate these findings in a larger sample.

**Supplementary Information:**

The online version contains supplementary material available at 10.1007/s10339-026-01347-3.

## Introduction

As we age, it becomes more difficult to learn new skills and knowledge, largely due to a decline in cognitive abilities associated with learning. A continuous age-related decline in cognitive abilities associated with learning, such as processing speed and working memory, begins in the second decade and accelerates as we become older (Salthouse 2010). Both processing speed and working memory are closely involved in storing and retrieving information, which are essential to successful learning. As such, learning new skills and knowledge becomes more challenging in older adulthood. However, learning in older age may be supported by collaborating with another person, lessening some of the challenges experienced by older adults (Wolfe et al. 2023).

Collaborative learning has been defined as cooperating and communicating with one or more people to learn a new skill or task, instead of learning alone (Dillenbourg 1999; Dixon and Gould 1998; Wolfe et al. 2023). Through learning together, partners can use their complementary or shared knowledge to further establish their collaboration (Canham et al. 2012). The topic of collaborative learning has recently experienced a revival and is being explored using various designs such as comparing older adults to younger adults (Crompton et al. 2022; Derksen et al. 2015; Rodrigues et al. 2021; Yoon and Stine-Morrow 2019), examining whether familiarity between partners affects learning (Crompton et al. 2022; Gould et al. 2002; Rodrigues et al. 2021; Xie 2011), and how the benefits of collaboration differ between learning tasks (Wolfe et al. 2023). Overall, successful learning through collaboration does not appear to depend on a pre-existing relationship between partners (Crompton and MacPherson 2019; Wolfe et al. 2025) and learning collaboratively is most beneficial when using novel, rule-based tasks, while the additional benefits of learning together are limited when using longer-duration training programs (for a review see Wolfe et al. 2023). Despite renewed attention to the potential benefits of collaborative learning in older age, there is little research on whether learners can achieve similar benefits from learning with a computer system as with a human partner. As such, the present study directly compared learning with a perceived human versus a voice-based computer system.

Interaction with voice-based systems, such as Amazon’s Alexa or Apple’s Siri, is a part of everyday life for many people (Zierau et al. 2023). As older adults frequently experience vision and motor difficulties, as well as a lack of familiarity with new technology (Fletcher-Watson et al. 2016), older adults may find using voice-based systems more accessible than computers, tablets, and smartphones (Upadhyay et al. 2023), and these systems are being increasingly adopted by older adults (Brewer et al. 2022). Voice-based systems and voice assistants, such as Amazon’s Alexa and the Google Nest, can support older adults to manage daily activities, social engagement, and health independently (Orlofsky and Wozniak 2022; Upadhyay et al. 2023), as well as casual conversation and “small talk” (Pradhan et al. 2020; Kim and Choudhury 2021). However, though older adults have reported enjoying using these systems (Orlofsky and Wozniak 2022), they often do not fully utilize the range of features of voice-based systems (Sin et al. 2022).

Most research into the use of voice-based systems in older adults has used interviews or focus groups to explore their experiences of using these systems and to identify barriers to their use (Pednekar et al. 2023). While this is important, it is also crucial to know how older adults process, retain, and recall information that they learn with voice-based systems (Crompton and MacPherson 2019). Our understanding of the cognitive processes that underlie social interactions with voice-based systems are relatively limited (Cross and Ramsey 2021).

Our social expectations of computers (and voice-based systems) are different to our social expectations for humans (Cross et al. 2016; Heyselaar et al. 2023), and our beliefs about a system’s agency and “human-ness” can affect how we perceive their behaviours (Klapper et al. 2014; Cross et al. 2016; Darda and Cross 2023), interact with them (Stenzel et al. 2012; Caruana et al. 2017) and learn from them (Crompton and MacPherson 2019). Our beliefs about a system’s agency affect how we engage with them on a joint task - for example, if a system is described as being “human-like”, participants are more willing to share a task load with it (Wahn and Kingstone 2021). A number of factors, including voice (Kim et al. 2021; Seaborn et al. 2021), conversational dialogue (Völkel et al. 2021) and expressions (Feine et al. 2019; Paradeda et al. 2016), can make the system more human and change the way that users experience it.

Very little is known about how older adults collaborate with computers, including how they might learn collaboratively together, and whether beliefs about agency may play a role in this. Crompton and MacPherson (2019) adopted a Barrier Task paradigm (Duff et al. 2006), which assesses visuospatial memory, an aspect of cognition which declines with age (e.g., Boone et al. 1993; Ostrosky-Solis et al. 1998; Gallagher and Burke 2007). They assessed how older adults interacted and learned with a system that they believed was a human, and with a system they believed was a computer. Traditionally, the Barrier Task involves two people working together to match abstract tangram cards based on referential labels (for example, “the one that looks like a spiky plant”) to locations on a grid. One person in the pair describes the items to the other person; their work spaces are hidden from one another’s view by a barrier. Over repeated trials, the labels used by directors to refer to specific items become simplified and shorter as the pair reach a “common ground” (Clark and Wilkes-Gibbs 1986; Glucksberg and Krauss 1967; Yule 1997). Rather than partners having shared referential knowledge to match identical labels, the pairs search to have an “acceptable” understanding of one another.

Crompton and MacPherson (2019) used a Wizard-of-Oz (WOz) paradigm (Green and Wei-Haas 1985) to study the effect of perceived interlocutor on collaborative learning efficiency and accuracy. In a WOz study, participants are systematically deceived about the nature of the system they interact with, and informed about the deception at the end of the study. In Crompton and MacPherson’s study, participants collaborated with a computer system on the Barrier Task. In half of the trials, they were told they were interacting with and being directed by a human (actually a computer using natural speech responses), and in the other half, by computer (a computer using synthetic speech responses). Participants who thought they were interacting with a computer were less accurate on learning trials, changed their answers between trials more frequently, and were less accurate on delayed recall of the tangram card descriptions after one hour, compared to the human condition. However, while the Barrier task is often used in collaborative learning research, this form of interaction is rare in everyday interactions.

As voice-based systems become increasingly ubiquitous (Zierau et al. 2023) and touted as a way to support aging in place for older adults (Cuadra et al. 2023), it is crucial to determine whether beliefs about agency affect how older adults learn from these systems. If older adults believe that their system does not have agency, they may learn and recall information from it less accurately (Crompton and MacPherson 2019). There has been very limited research in this area, and it is unclear whether these agency effects on collaborative learning and later recall would be replicated when using a different learning task. Here we used a Map Task paradigm (Anderson et al. 1991) that is considered an ecologically valid measure of real-life communication, as planning and navigation are skills regularly used in daily life to complete tasks such as learning a route to a new location (Anderson et al. 1991; Bangerter and Clark 2003; Mori et al. 2011; Pardo et al. 2013). In the Map Task, pairs must work together to plan and execute a route, doing so through natural conversation. Using this task allowed us to study collaborative learning in a more ecologically valid way than the Barrier Task and added novel and important insight into collaborative learning in aging.

As well as being a more ecologically valid measure of real-life communication, the Map Task assesses aspects of spatial cognition that typically deteriorate with age (Fernandez-Baizan et al. 2020; Coughlan et al. 2018; Daugherty and Raz 2017; Hill et al. 2023; León et al. 2016). Our ability to navigate in complex environments involves storing and recalling information about one’s environment and our positioning in it, updating this information, manipulating it over time, and devising navigation strategies (Daugherty and Raz 2017; Wolbers and Hegarty 2010). As these processes are considered to be cognitively demanding, and more so for older adults, younger adults have typically performed better than older adults on spatial memory tasks (Hill et al. 2023; Klencklen et al. 2012).

The current study attempted to determine whether the differences in older adults’ collaborative learning efficiency and accuracy, which have been found to depend upon beliefs about the system’s agency (i.e., a human versus a computer) when using the Barrier Task, were also found using a Map Task. Wykowska et al. (2014) argue that, when participants perceive that they are interacting with a human partner, they understand the behaviours to be created by an intelligent mind with intention. Conversely, when participants interact with computers, they assume a “design stance” where behaviors are created by an engineer; therefore, participants change how they communicate with the system. Additionally, older adults are less likely to be familiar with computer-based systems, leading them to perform more poorly on computerized versions of tasks compared to non-computerized versions (Kosowicz and MacPherson 2017). Therefore, if older adults still perceive systems as being ‘a computer’, they will interact with them differently, and may not learn as accurately with them. If this effect holds across two learning and memory tasks that emphasise collaborative social interaction, which is crucial to some technology-based support for age-related decline, it is more likely that it will generalise to other types of collaboration.

We focused on older adults as difficulties with learning and memory are more prevalent in this age group and therefore benefits of collaboration may be more likely. We measured older participants’ (a) efficiency at completing a collaborative learning task; (b) accuracy at completing the task; and (c) accuracy at independently recalling the collaboratively learned information after a delay of one hour and one week. Based on previous findings, we tested the following hypotheses:

1. Participants would become faster to complete the task over trials;
2. Participants would be faster completing the trials with the human partner compared with the computer partner;
3. Participants would be more accurate when completing the trials with the human partner compared with the computer partner;
4. Participants would be more accurate when recalling the routes (at immediate and delayed recall) learned with a human compared with the computer partner.

## Methods

### Participants

Twenty-four older adult participants aged 66–79 years (mean = 72.21, SD = 3.55; 17 females; two left-handed) were recruited through the University of Edinburgh Volunteer panel to attend a 90-minute session on learning together. They were remunerated for their time. Participants had an average of 16.29 years of full-time education (SD = 2.96, range = 11–23) and all were native British English speakers. All participants lived independently and none of them had any history of the neurological or psychiatric disorders listed in the Wechsler Adult Intelligence Scale–III UK (WAIS-III UK) and Wechsler Memory Scale-III UK (WMS-III UK) selection criterion (Wechsler 1997a; Wechsler 1997b). Ethical approval was granted by the School of Philosophy, Psychology, and Language Sciences (PPLS) Research Ethics Committee, and all participants provided written informed consent.

### Procedure

#### Cognitive tests

All participants individually completed a battery of cognitive tests to determine whether they performed within the expected range for same-aged cognitively healthy older adults. The Test of Premorbid Functioning (Wechsler 2009) provided a measure of full-scale IQ. Participants were required to read aloud a list of 70 irregularly pronounced words due to their irregular grapheme-phoneme translations. In the Rey Osterrieth Complex Figure Test (Meyers and Meyers 1995), which assesses immediate and delayed visuospatial memory, participants were required to copy a complex geometric figure and then redraw the figure from memory 2 mins and 25 mins later. Higher scores indicated better episodic memory. Participants were awarded a maximum score of 36 for each of the three drawings (copy, immediate and delayed recall). The WAIS-IV Digit Span Subtest (Wechsler 2008) assessed working memory capacity and required participants to listen to a sequence of numbers and then recall them back in the same (forwards), or reverse (backwards) order, or reorder and recall the numbers in ascending order, starting with the lowest number (e.g., 8, 3, 5 into 3, 5, 8; updating). The final score was the total number of correctly recalled trials, out of a maximum possible score of 16, for each condition. Higher scores indicated better working memory capacity.

#### The Computerised Map task

To compare how participants behaved and performed when they believed they were interacting with a human or a computer, a Wizard-of-Oz (WOz) simulation (Green and Wei-Haas 1985) was created using Python (Tullo and Crompton 2018). In this paradigm, the participant believed that they were interacting with a computer system that automatically responds to their input, when they were in fact interacting with a system that was being manipulated or semi-manipulated by a human operator. A WOz paradigm creates the illusion of an intelligent computer system, while the human operator intercepts the user’s input and manipulates appropriate responses in real-time, often through using keyboard shortcuts (Green and Wei-Haas 1985). Using a WOz set-up allowed the creation of two directly comparable human and computer conditions where every aspect of the interaction was identical and constant, with the only manipulations being participants’ beliefs about their study partner, and whether information was presented using natural or synthetic speech. The natural speech used in the human condition was provided by a female speaker with a Scottish accent. The synthetic speech used in the computer condition was provided by the Festival Speech Synthesis System (2026) and a female voice (“Nina”) to match the gender of the natural speech voice. The command-line utility Normalize (2026) was used to equalize the sound levels across all sound files.

**Fig. 1:**
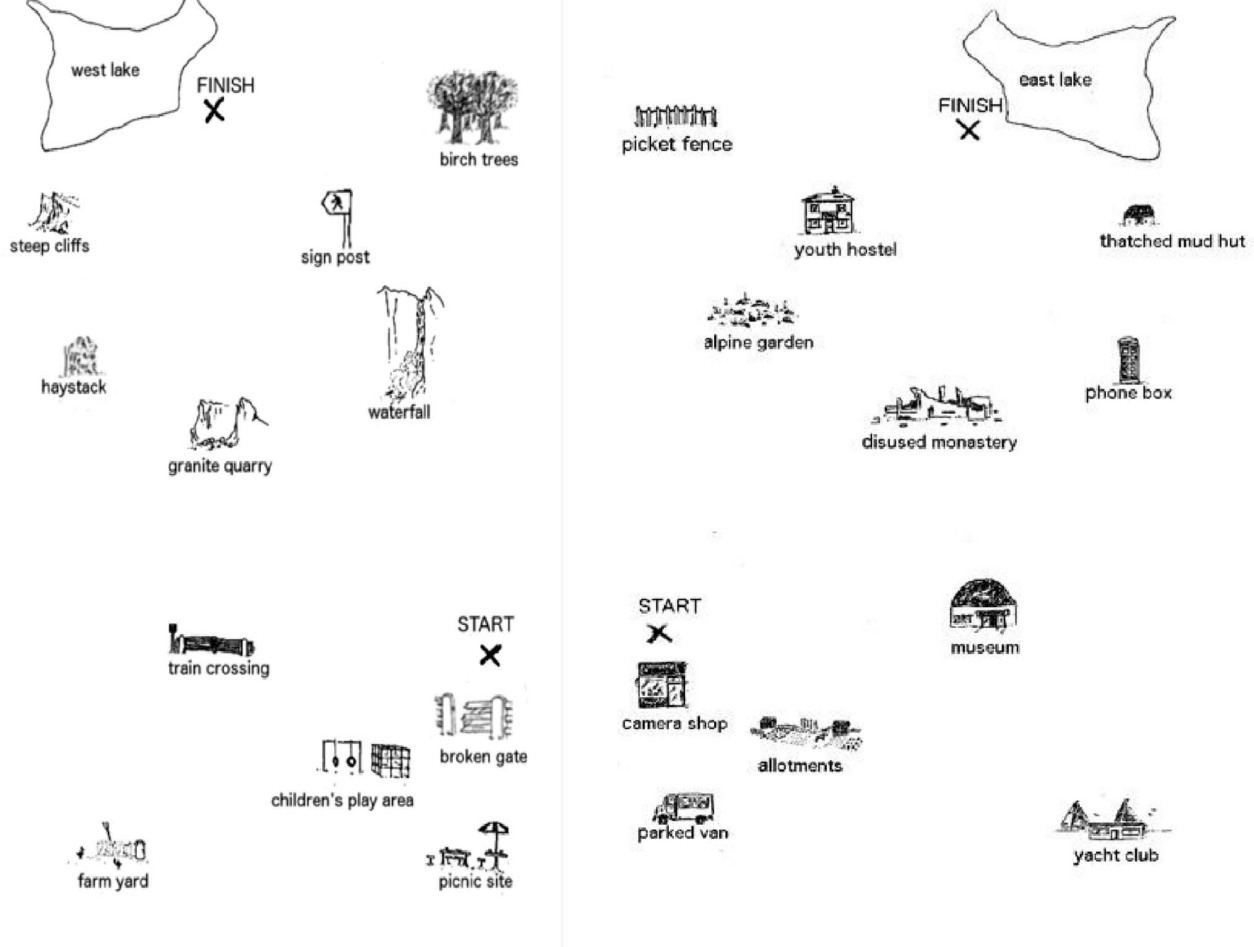
Map A (Left) and Map B (Right) of the adjusted Map Task paradigm from The Human Communication Research Centre (2022)

The Computerized Map task was based on the Map task paradigm previously used in human interaction studies without the manipulation of agency (Anderson et al. 1991; The Human Communication Research Centre, 2022). In the Map task, participants work collaboratively to recreate a route on a blank map showing several landmarks (see Fig. 1). Through conversation, pairs must establish the correct route from start to finish. Participants are not allowed to stand up, view or interact with the other person’s map, and must convey information through speech, gestures, and expressions. The original maps 0 and 4 from Anderson et al. (1991) were chosen as each route contained the same number of landmarks, and they were inverted images of the same route pattern, and therefore were matched for difficulty. The maps were edited so the landmark labels were replaced with clearer, computerised text to make reading easier for older adults, an even number of landmarks were presented on each map, and some landmarks were replaced with alternatives from other maps to ensure that the maps had the same amount of one-, two- and three-word landmarks on them. The administration of the Map task was based on that of Anderson et al. (1991) to elicit spontaneous oral production through the participant taking on the role of direction-follower (for a review of administration of the Map task see Berrios et al. 2023). The study partner, who was the direction-giver, was either a perceived computer or a human research assistant (in both cases a WOz simulation but using natural language). At the start of the session, participants were told they would be completing a route map task twice, once with a research assistant named Rosie, and once with a voice-based computer system. Before completing the task with Rosie, participants were told that she was in an adjacent lab, and they would be communicating with her using headphones and microphones. Before completing the task with the computer system, participants were told that they would be interacting with a state-of-the-art voice-based computer system that would recognize what they said and reply verbally, and that they should communicate using the microphone and speakers. The system was not referred to as an “artificial intelligence” as the focus was on participants’ reaction to computer systems in general, not to artificial intelligence in particular. In reality, in both conditions participants were interacting with the WOz system run by the experimenter in an adjacent room.

In all trials, the participant received instructions from the Describer (i.e., the research assistant Rosie or the voice-based computer system). Each trial included ten directions to guide participants to the correct route; one direction related to each of the twelve landmarks on the map. The directions were intentionally vague and did not include an essential piece of information that would be required to accurately complete the task: for example, in Map A, the Describer instructed the participant to go over to the left side of the monastery, while not telling them whether they should pass underneath it, or over the top of it. This was done to encourage participants to interact with the Describer, as a dependent variable in this study was the number of turns taken in the interaction. Participants were given the following instructions, which were developed for the current study:

“Rosie doesn’t have a copy of the map, and only has a list of the instructions describing the route. She will be reading from a script she has been given to describe the route to you. She also has some additional information about the route, as the information that she gives you may be deliberately vague and therefore you need to ask for additional directions to draw the route correctly. You are scored on how correct your drawn route is, and not on the speed with which you complete the task, so take your time to get it right. You should go around the landmarks and not draw through them.”

Participants were therefore encouraged to ask for additional information about the route. Numerous responses were available within the system and activated by keystroke entry to the WOz system (see Supplementary Tables 1 and 2). If participants asked Rosie or the voice-based computer system questions that did not have scripted responses, ad-hoc responses were selected such as, “I’m not sure, I don’t have that information in my script”.

Participants completed the Map task three times per condition (perceived human and computer), each time with a new copy of the same map and same route. Three trials were used in each condition as this provided participants with multiple exposures, which should lead to successful encoding of the route information. More trials would likely lead to ceiling effects in later recall of the routes. In addition, participants used a different version of the map for each condition (see Fig. 1), with the two map types and conditions counterbalanced across participants (see Supplementary Table 3).

Participants’ performance on the Map task was assessed using the accuracy of their drawn route. The scoring system for this variable was adapted from the scoring used by Anderson et al. (1991). Both routes had twelve landmarks, and a maximum of three points would be given per landmark, depending on the following: one point was given if the landmark was passed in the correct chronological order, one point for passing the landmark on the correct side of the map, and another point for passing by the landmark at an appropriate distance (e.g., it did not count as a correct pass if the line was more than half of the average of the landmarks’ width or height away from the landmark). Overall, participants were able to achieve a maximum score of 36 per trial.

In line with our previous collaborative learning work (Crompton and MacPherson 2019; Crompton et al. 2022; Wolfe et al. 2025), we also measured participants’ time to complete each trial in seconds, and the number of turns within each trial. We characterized a turn as an utterance produced by the participant, with the end of a turn characterized by a change in active speaker (i.e., the Describer giving a new direction).

Participants were told to complete the Map task as accurately and quickly as possible but were not explicitly told to concentrate on one performance measurement over another.

#### Delayed recall of the Computerised Map task

After completing the Computerized Map task, participants were asked to re-draw the route that was described to them during the study. Participants were prompted to do so immediately, at one-hour post-completion of the task, as well as a week later. The one-hour delay condition was completed in the laboratory, during which the cognitive tests were performed, and the one-week delay was completed at home. Participants were sent a blank copy of the map in the mail and asked to open it and complete the route one week after their initial learning session. Participants returned their completed maps via post. The delayed recall condition was scored in the same way as the learning trials, with a maximum score of 36 for each map, with a high score indicating greater recall accuracy.

#### Computerised Map task user questionnaire

Immediately after each of the human and computer conditions of the Map task, participants completed a short, verbally administered, six-item questionnaire, which was developed for this study, to compare participants’ view of interacting with a human or computer partner. Participants orally provided short verbal responses before rating each question on a 1–7 Likert scale where 7 represented a higher score and a better interaction. The questions were:

1. How difficult did you find the task?
2. How confident are you that you have drawn the correct route?
3. How easy was the system/Rosie in the other room to interact with?
4. How was the quality of the descriptions of the route that the system/Rosie provided?
5. Could the system/Rosie understand what you said?
6. Could you understand the system/Rosie?

After completing this questionnaire, participants were informed of the true nature of the study and asked whether they believed they were interacting with Rosie, a research assistant in an adjacent room.

### Data analysis

All data were analysed using R (R Core Team, 2024), version 4.4.1, and RStudio (RStudio Team 2024), version 2024.04.2. No data were removed from our analysis. To investigate Map task accuracy and efficiency (of which variables are the time to complete, and the number of turns taken), we used linear mixed effects models, implemented using the lme4 package (Bates et al. 2015). Model building was done using a forward stepwise approach, starting with a null model and then adding predictors to alternative models in the following order: Condition, Trial and then the Condition x Trial interaction. All models included the random effects structure of (1 + Condition|Participant). This allowed us to take into account the individual variability across participants and conditions, and enabled the model to consider differences that are not explained by fixed effects. Fixed effects of Condition (human versus computer) and trial (1, 2 and 3) were entered as main effects and then as interactions. Each alternative model was compared to the previous model using ANOVA to determine whether addition of the relevant fixed effect significantly improved model fit. Where independent variables such as condition improved model fit, though not significantly, they were still included in the model as main effects, as they were key manipulations in this study and the influence of which cannot be ascertained if they are not included. Models were standardised using the ‘standardize’ function of the arm package version 1.14-4 (Gelman et al. 2024). Standardised models centred all binary and continuous variables, and scaled continuous variables by 2 standard deviations. This meant that the model outputs were on the same scale, and continuous variables could be compared with binary variables. The degrees of freedom and associated p values were ascertained using the package lmerTest (Kuznetsova et al. 2017), version 3.1-3, to compute the Satterthwaite approximation. Further details of the best fitting models can be found in the Results section. The threshold for statistical significance was |t| > 1.96 where the null hypothesis was rejected for any t-value above 1.96. Smaller sample sizes may require adjusting this threshold depending on the degrees of freedom. The conditional *R*^2^ for the best fitting models was calculated, which is the variability attributed to both the random and fixed effects. The marginal *R*^2^ for each main effect, as well as their 95% confidence limits, were also calculated to determine the proportion of variability attributed to the fixed effects only (Nakagawa and Schielzeth 2013).

The same linear mixed effects model analysis was conducted to analyse the Map task delayed recall data, except the models included the (1 + Time|Participant) random effects structure. This analysis took into account the individual variability across participants and time to complete, and enabled the model to consider differences that are not explained by fixed effects. Fixed effects of Condition (human versus computer) and Delay (immediate, one hour and one week) were entered as main effects and then as interactions. Again, the conditional *R*^2^ and the marginal *R*^2^ and their 95% confidence limits were calculated.

To analyse the user questionnaire data for the human and computer conditions of the Map task, when the data were normally distributed, a repeated-measures t-test was conducted. When the data were non-normally distributed, a Wilcoxon Signed Ranks test was used. False Discovery Rate (FDR) was applied to reduce the likelihood of Type-I familywise error.

A sample size of 24 participants was planned, based on the time and resources available to complete the study. To allow independent replications, we provide the full dataset and our analyses scripts for the healthy older participants (10.17605/OSF.IO/6V2CM). No part of the study’s procedures or analyses was pre-registered prior to the research being conducted.

## Results

### Cognitive tests

Table 1 illustrates participant performance on the three cognitive tests. All participants performed within the expected range for same-aged cognitively healthy adults.

**Table 1:** Participant performance on standardised cognitive tests

	Mean	SD
Test of premorbid functioning (max = 135)	113.17	5.72
Rey Osterreith complex figure, immediate recall (max = 36)	20.50	4.21
Rey Osterreith complex figure, delayed recall (max = 36)	20.56	4.17
Digit span forwards (max = 16)	12.08	1.67
Digit span backwards (max = 16)	9.96	2.10
Digit span sequence (max = 16)	8.67	1.88

### Computerised Map task efficiency

**Fig. 2:**
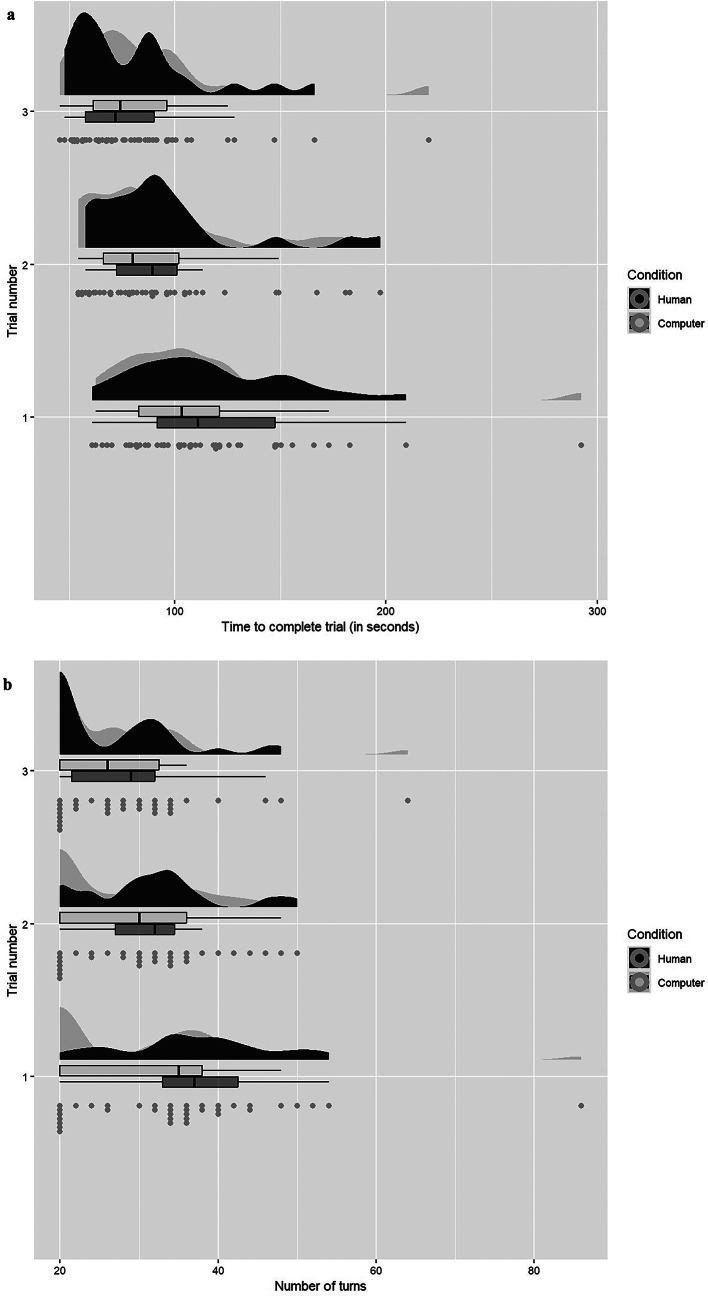
**a** Raincloud plot for time to complete learning trials 1–3 with human and computer partners. **b** Raincloud plot for number of turns taken during learning trials 1–3 with human and computer partners

For time taken during each trial, the best fitting model included a significant main effect of trial (*p* < 0.0001), indicating that participants became more efficient in later trials (see Fig. 2a; Table 2; Supplementary Materials Table 4). However, the main effect of condition was not significant. The conditional *R*^2^ was 82.9% (i.e., the total model variance), with the main effect of trial contributing to 11.6% of the variance and the effect of condition only contributing 0.2%. Therefore, much of the model variability resided in the random effects, with the main effect of trial having a medium effect. There was no interaction between condition and trial, suggesting that the improvement over trials was regardless of whether participants were performing in the human or computer conditions.

**Table 2:** Time taken: beta, standard errors, t-values, and marginal *R*^2^ for fixed effects

	Estimates	Standard error	t-value	95% CI	Marginal *R*^2^	95% confidence limits
*Fixed effects*						
Condition (Computer)	– 3.37	4.05	– 0.83	[– 11.31, 4.57]	0.002	[0.000, 0.044]
Trial	– 26.49	2.69	– 9.84**	[– 31.76, – 21.21]	0.116	[0.038, 0.225]

* t > 1.96, ** t > 2.58

For number of turns taken, the best fitting model included a main effect of trial (*p* < .0001), indicating that participants used significantly fewer turns as trials progressed, with no significant difference between the human and computer conditions (see Fig. 2b; Table 3; Supplementary Materials Table 5). The conditional *R*^2^ was 80.0%, with the main effect of trial contributing to 7.7% of the variance and the effect of condition only contributing 1.0%. Therefore, much of the model variability resided in the random effects with only a small effect of trial on number of turns. Again, there was no interaction between condition and trial, suggesting that the difference in number of turns taken with human and computer partners remained stable over trials.

**Table 3:** Number of turns taken: beta, standard errors, t-values, and marginal *R*^2^ for fixed effects

	Estimates	Standard error	t-value	95% CI	Marginal *R*^2^	95% confidence limits
*Fixed effects*						
Condition (Computer)	– 1.97	1.07	– 1.84	[– 4.07, 0.12]	0.010	[0.000, 0.068]
Trial	– 5.60	0.76	– 7.37**	[– 4.33, – 2.51]	0.077	[0.015, 0.176]

*t > 1.96, ** t > 2.58

### Recall accuracy

**Fig. 3:**
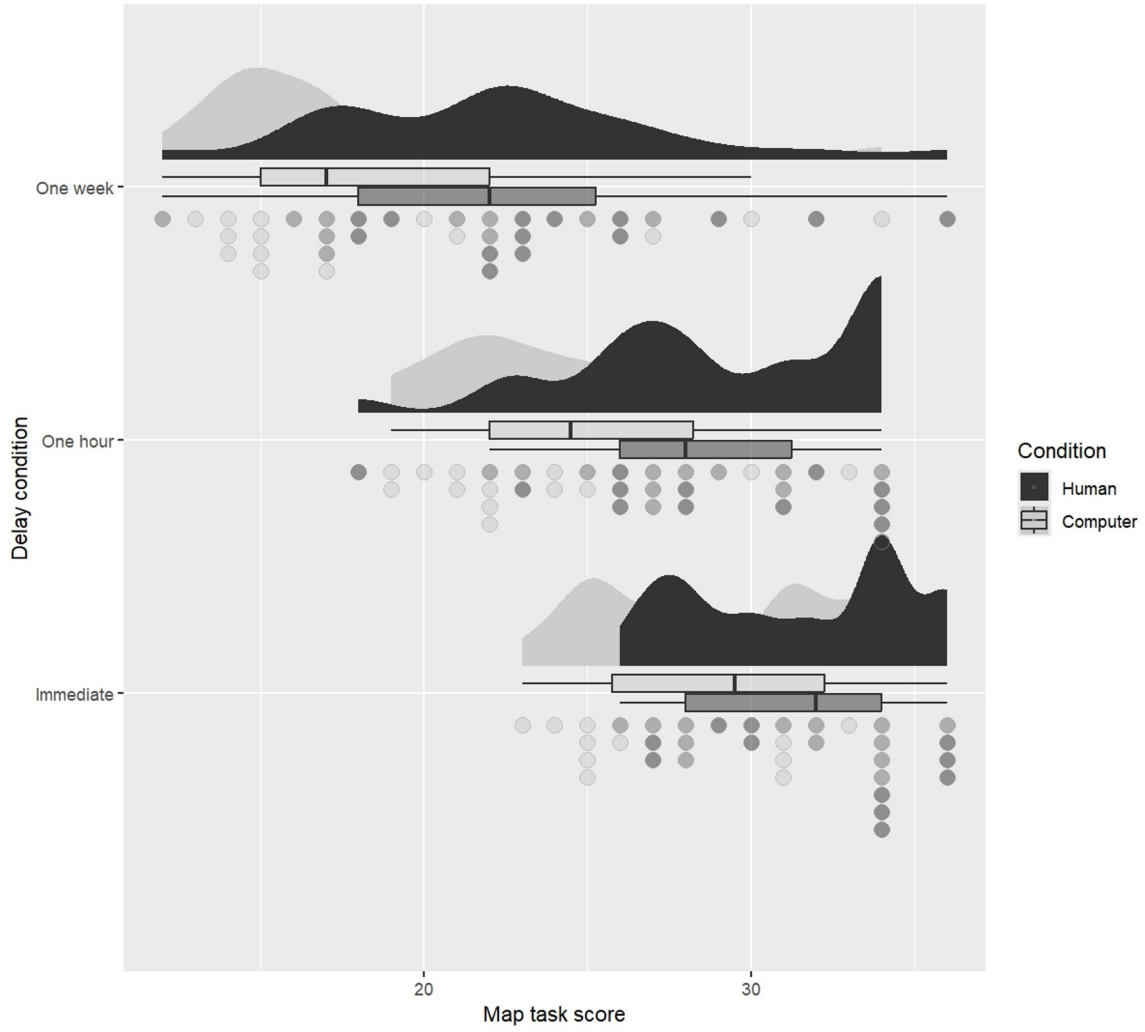
Raincloud plot for recall scores at immediate, one hour and 1 week recall for routes learned with human and computer partners

For recall accuracy, there was a significant main effect of condition (*p* < .0001), indicating that participants recalled the routes learned with the human partner significantly more accurately than the routes learned with the computer partner. There was also a significant main effect of delay where both recall after one hour and one week was poorer than immediate recall (*p* < .0001), indicating that the amount of information recalled by participants significantly declined between immediate, one hour, and one week recall for both conditions (see Fig. 3; Table 4; Supplementary Materials Table 6). The conditional *R*^2^ was 64.9%, with the main effect of condition contributing to 9.9% of the variance and the effect of delay contributing 43.8%. Therefore, there was a large effect of delay over and above the random effect variance and a medium effect of condition. There was no interaction between condition and delay.

**Table 4:** Recall accuracy: beta, standard errors, t-values, and marginal *R*^2^ for fixed effects

	Estimates	Standard Error	t-value	95% CI	Marginal *R*^2^	95% Confidence Limits
*Fixed effects*						
Condition (computer)	– 2.99	0.64	– 4.67**	[– 4.24, – 1.73]	0.099	[0.027, 0.204]
Delay (1 h)	– 3.67	0.75	– 4.89**	[– 5.14, – 2.20]	0.438	[0.332, 0.545]
Delay (1 week)	– 9.65	0.75	12.85**	[– 11.12, – 8.17]	0.438	[0.332, 0.545]

* t > 1.96, ** t > 2.58

### Computerised Map task user questionnaire

There were no significant differences in terms of task difficulty or the understanding of the participant. This meant that participants rated the natural and synthetic voices used in the human and computer conditions similarly in terms of task difficulty and ease of understanding (see Table 5). However, there were significant differences in all other aspects of the interaction. Participants rated the computer partner’s ability to understand the participant as poorer and they were significantly less confident in the accuracy of their routes. They also rated the human as significantly easier to interact with. Participants perceived the description of the route as significantly poorer quality with the computer, even though the content of the route descriptions provided by the human and computer was identical.

On completion of the questionnaire, participants were informed that they had been interacting with a human using a WOz computer system in both conditions and asked if they had been convinced of the human and computer conditions during their session. Deception was successful for all participants, as none of the participants reported being aware that Rosie was not a human research assistant.

**Table 5:** Likert scale self-report responses on user questionnaire for aspects of the interaction during the Computerised Map task with human and computer partners

	Human	Computer
Task difficulty	6.42 (0.50)	5.88 (1.23)
Task confidence	6.42 (0.58)	5.21*** (1.14)
Interaction ease^a^	7.00 (0.00)	6.38** (0.88)
Quality of description^a^	6.67 (0.56)	5.33*** (1.24)
Understanding of participant	7.00 (0.00)	6.88 (0.61)
Understanding of system	7.00 (0.00)	6.92* (0.28)

^a^Wilcoxon Signed Ranks test; *p < .05; **p < .005; ***p < .0001

## Discussion

In this study, older adults completed a collaborative Computerised Map task and their learning and recall performance were assessed. They completed the task in two conditions of a WOz paradigm: in one, participants believed they were interacting with a human research assistant; in the other, participants believed they were interacting with a computer program. The verbal content was identical in both cases, with the human condition using natural speech and the computer condition using synthetic speech. This paradigm allowed us to examine the effect of agency beliefs on human interactive behaviors, on learning, and on recall after a delay. We also examined participants’ perceptions of the skill of the computer program and perceptions of their own performance when interacting with a computer program compared with a partner they believed to be human.

Participants were faster at completing the Computerised Map task in later trials compared to earlier trials in both the human and computer conditions. This is a common finding in collaborative learning tasks (e.g., Derksen et al. 2015; Crompton and MacPherson 2019; Crompton et al. 2022) and indicated that participants became more familiar with the Map task route over repeated trials (Wolfe et al. 2025). There were, however, no differences in efficiency between the human and computer conditions. Participants did not significantly differ in the amount of time or the number of turns they produced to complete the task with a human partner compared with a computer partner. The narrow range of values indicated by the confidence limits for the main effect of Condition suggests that learning with a human or computer partner has little or a small effect on the models. However, we would strongly recommend that future research replicates our study in a larger sample to confirm these findings.

The lack of an interaction between Condition and Trial seems to speak against the notion that agency belief effects are stronger when a task is less familiar and presents more task ambiguity. In younger adults, as a user becomes more familiar with a system, the cognitive demands of the task reduce, and the user’s comfort and engagement with the system increase (Wang and Sun 2025). However, in our small sample of older adults, time did not reduce variability in Map Task performance, and did not explain the lack of differences between the human and computer conditions. One shortcoming of our study was not measuring our participants’ experience with technology or administering a technology self-efficacy measure. These are two important potential moderators that should be further explored in future work; individuals with greater knowledge and exposure to technologies may have more positive perceptions of learning with technology and, in turn, demonstrate higher accuracy and efficiency.

These findings do not support our previous research where older adults engaged in a collaborative Barrier task with perceived human and computer partners using a similar WOz paradigm (Crompton and MacPherson 2019). In that study, participants were slower at completing initial trials with a human, but were significantly more efficient by the end of the trials. Participants were also more accurate when they believed they were interacting with a human than when they believed they were interacting with a computer. When interacting with a computer, the best strategy is still to find a terminology that the computer will understand. Essentially, rather than finding the “common ground”, participants should build an appropriate mental model of the computer, since it may be difficult for the computer to adapt to the human. When interacting with a human, on the other hand, it is possible to truly negotiate “common ground”, because the human interlocutor is expected to be willing and able to adapt.

The instructions given for the Map Task, on the other hand, include enough information for participants to complete each stage (for example “go past the barn”) except for a missing piece of information that participants had to request in order to complete the route correctly (for example “go past the barn…on the left”). As most of the missing information had binary options (e.g., left or right, above or below), it was possible to correctly navigate around each of the landmarks by guessing the directional detail missing from the core instructions without requesting additional information from the perceived human or computer, and educated guesses are a valid interaction strategy for both human and computer interlocutors. Therefore, participants performed the task with similar accuracy regardless of the perceived partner. Certainly, the lack of a significant main effect of condition for the number of turns suggests that older adults were not more likely to seek help in one condition compared to the other. This finding should be replicated in a larger sample of older adults.

The reasons for the different findings between participants’ interactions with the perceived computer and the perceived human, depending on the task adopted, need further investigation. For example, a direct comparison between the Barrier Task and the Map Task within the same study would allow us to infer differences between the tasks. Wolters et al. (2009) observed that while some older adults treated a simulated appointment scheduling system like an interactive voice response system, others treated it more like a human. Similarly, Nass and Brave (2005) found that when adults engage with computer systems, they often use conversation strategies and mental models that are more suited to human-human interaction. It is possible that participants engaged with the computer on a social level (i.e., they sought additional information not pertinent to the task or attempted to make small talk) because they considered the computer capable of truly responsive interaction (Rietz et al. 2019).

Future research may focus not just on the perceived agency of the partner but also their perceived helpfulness, for example whether they give active and engaged feedback. For older adults, beliefs about agency may be affected by assessment of and feedback on their performance (Metcalfe et al. 2010). It would be interesting to examine performance on this type of task with humans and computers that vary in the degree of assistance they give to examine whether such assistance behaviours affect agency. Additionally, given the jump in naturalness and the substantially increased prevalence of dialogue systems and chatbots, it would be of interest to see whether future collaborative learning studies will have similar findings, and to what extent attitudes to and experience with technology affect participants’ mental model of the spoken dialogue interface. Future work may examine whether this effect persists using other technological settings commonly used by older adults, for example, app or browser-based tasks. Moreover, research using other technological settings may facilitate our understanding of the mechanisms underlying the impact of agency beliefs on interaction, learning and memory.

### Immediate and delayed recall

Participants recalled the route learned with the human with more accuracy than those learned with the computer, immediately, at one hour and one week after the study session. This aligns with prior findings on human-computer interactions; for example, participants demonstrated higher recall for list items that were supposedly recalled by another participant compared to a computer, suggesting that processing information in a social manner improves recall performance compared to nonsocial processing (Reysen and Adair 2008). It also aligns with work using the Computerized Barrier task (Crompton and MacPherson 2019), in which participants recalled more descriptive labels learned with the human partner one-hour post-study. Given that there were no differences in the engagement of participants with the perceived human or computer while learning the task, this suggests that more accurate delayed recall is not due to higher engagement with the learning materials when interacting with a perceived human partner, but this finding needs replicated. With older, less natural text-to-speech systems, it is possible that information provided using synthetic speech is recalled less accurately compared to natural speech (Luce et al. 1983; Smither 1993; Thomas et al. 1989; Wolters et al. 2007, 2015). However, synthetic speed intelligibility has increased rapidly over the intervening years, with most cutting-edge research systems being almost indistinguishable in terms of intelligibility (Perriton et al. 2025).

### Participants’ perceptions of the Wizard-of-Oz paradigm

The participants in this study rated the natural and synthetic voices as being similarly easy to understand and similar in terms of difficulty. However, participants reported finding the computer partner less able to understand them and the given descriptions as less accurate and feeling less confident about their learning performance. This finding is similar to that of Crompton and MacPherson (2019), in which the Barrier task was used to investigate human-computer interaction. Those results showed that participants found the human and computer similar in terms of the ease of understanding their speech, but the experience of learning the Barrier task was considered less effective when working with the computer. It may be that older adults’ perception of computer-based information is affected by negative attitudes to technology (Mitzner et al. 2019), the perceived lack of usability of technology (Pirhonen et al. 2020), low technology self-efficacy (Wilson et al. 2021), and stereotype threat (Mariano et al. 2020) leading to less confidence about their performance. In addition, the perceived ability of a collaborative learning partner affects one’s motivation, with less able partners leading to poorer learning outcomes (Jones and Issroff 2005). As such, participants’ perception of lower quality information given by the computer could have impacted their motivation to learn, resulting in poorer longer-term learning outcomes compared with the human trials. In future work, older adults’ ability to learn from computers should be investigated within the framework of existing technology acceptance and use models, such as UTAUT (Macedo 2017). These models integrate a variety of relevant factors such as attitudes to technology, technological anxiety, motivational factors, contextual issues, experience with technology, level of education, and socio-economic background.

People’s perception of synthetic speech is influenced by familiarity. Though machine-created speech is becoming more commonplace (through applications such as Apple’s Siri or Amazon’s Alexa), older adults are less likely to use these systems and are less familiar with synthetic speech compared with younger generations (Pradhan et al. 2020; Orlofsky and Wozniak 2022). The lack of familiarity with synthetic speech may have resulted in participants’ perception of the computer as being less useful compared to the human. Future work may utilize this type of task with a range of age groups to examine whether younger and older adults are affected by agency beliefs in the same way, and whether similar factors affect how successful younger and older adults are when learning from perceived human and computer partners.

Overall, participants engaged similarly with their human and computer partner, but recall was more accurate when the information had been provided by the human, compared to the computer. Participants’ negative perceptions about the computers-provided information and computers themselves may have driven this effect.

### Limitations

We acknowledge some limitations of our study. Firstly, we may have missed some small effects in the data due to our modest sample size. In addition, our study focused on older adults’ learning of the Map Task and did not include a younger age group; during pilot testing, it was evident that none of the younger participants believed in our manipulation that a human partner was present (i.e., a human research assistant was in an adjoining lab), and instead realised that a computer program using natural speech recordings was being used. Therefore, we cannot confirm whether the same results would be found in younger adults if the manipulation was believed. We would hypothesise that, using a more sophisticated paradigm, younger adults would show similar results given that our previous collaborative learning work with human partners has shown that participants became more efficient at completing the Map Task over time, regardless of age (Wolfe et al. 2025). Moreover, our sample was predominantly female and highly educated and research with a larger and more diverse sample is needed to understand the generalisability of these findings. Additionally, the Computerised Map task had a fairly narrow range of accuracy scores. As differences in accuracy across the learning trials were approaching statistical significance, more research using a task which elicits a wider range of accuracy scores may be useful to examine whether there are differences in learning with perceived human and computer partners. As our study was designed to examine how agency beliefs affect interaction, learning and memory, it was necessary to use natural and synthetic speech to create believable human and computer partners. While studies on early speech synthesis systems showed that synthetic speech placed heavier demands on memory, which may impact older adults (Luce et al. 1983; Smither 1993), a more recent study using the same speech synthesis system showed no difference in memory for medication names compared to human speech (Wolters et al. 2015).

Other limitations relate to partner context. In our human condition instructions, we highlighted to participants that Rosie, the research assistant, could provide limited information and the instructions were intentionally vague to encourage interaction. This was necessary to convince older adults that they were interacting with a human research assistant in an adjoining lab. However, it was not necessary to state this in the instructions for the computer condition. One could argue that a human partner providing limited information is a relatively contrived context. Additionally, we did not have participants rate the amount of agency they perceived their human or computer partner to have. Indeed, as technology has become more interactive, tools such as voice-based systems have become more commonplace. Perceived agency of technology may also be shifting due to the emergence of artificial intelligence tools with natural language interfaces such as ChatGPT in 2022. Future studies should focus considerably more on agency and decision-making abilities and what technology can afford us in addition to studying interactions with the technologies (Sundar 2020). Finally, we did not create an overtly social computer condition, for example one which appeared chatty and verbose, which may have affected older adults’ willingness to socially engage during the computer condition. The perceived sociality of the computer plays a role in how users interact and learn from them; with higher perceived humanity related to increased interaction with a computer (Vardoulakis et al. 2012; Schuetzler et al. 2020; Rietz et al. 2019). Future research may want to investigate whether using a more overtly “useful” and social computer system affects older adults’ performance on the task, as well as their perception of their own performance and their perspective of the computer itself. Finally, our data collection methods did not facilitate examination of the linguistic content of the interaction. Recording and transcription of these interactions would allow us to examine how people interact, and whether language use differs based on agency beliefs. This would also allow comparison with Wolters et al.’s (2009) findings that some older adults used human-like interaction patterns with an automated system. However, despite these limitations, this study is one of the few studies that examines the effects of agency beliefs on interaction, learning, and memory in older adults, and indicates that perceptions of partner humanity play an important role on long-term recall.

### Implications

Speech systems are becoming increasingly prevalent throughout society and may promise innovative applications for aging in-place for older adults. In this study, we directly compared whether beliefs about a learning partner’s agency significantly affect the way in which older adults behave and learn, with results indicating that agency beliefs do have an effect. If older adults believe they are interacting with a computer, their memory recall is poorer. Given that this result has been successfully replicated for two different tasks, we suggest that it may well hold more generally; but of course, replication in a larger sample is needed. As beliefs about agency have an impact on how older adults interact with and learn from systems, researchers and software designers may want to take this into account when introducing learning materials using natural or synthetic speech to older end-users, as well as when they consider strategies for negotiating agency between the human user and the voice-based system (for example, integrating social elements into the user interface of voice-based systems, e.g., Sundar 2020). Research should explore how the wider social context of technology use may affect users’ interactions with systems to maximise usability and user outcomes.

## Conclusion

This study found no significant differences in how older adults collaborate and learn when they believe they are interacting with a human or with a computer. However, participants’ long-term memory recall was more accurate when learning with a human partner compared with a computer and so beliefs about agency affect how people remember information. Though participants reported that they found the human and computer similarly easy to understand, learning with the computer was rated as more difficult and they had lower confidence in their learning performance. Future research should investigate whether people’s perception of a computer partner and its instructions directly affect learning performance. These findings may inform future design of learning materials for older adults with a reliance on natural or synthetic speech, as well as future research on synthetic speech and its usability for learning in older age.

## Supplementary Information

Below is the link to the electronic supplementary material.

Supplementary file1
