# Supplementary material for: The impact of perceived human and computer interaction on collaborative learning: insights from the Map Task
Source: Cogn Process. 2026 May 27;27(3):703–18. doi: 10.1007/s10339-026-01347-3 (PMC13423963; doi:10.1007/s10339-026-01347-3)
Supplement: Supplementary file 1 — Supplementary file1 [file 10339_2026_1347_MOESM1_ESM.docx]

**Supplementary Materials**

Supplementary Table 1: Instructions given to participants during the Map task (Maps A and B), adapted from human-human interaction transcripts.

| **Landmark** | | | **Instruction** | | **Missing information** | |
| --- | --- | --- | --- | --- | --- | --- |
| **Set A** |  | | | | | |
| The camera shop | | | From the start you go down past the camera shop | | Which side of the camera shop to pass on (left) | |
| The parked van | | | You then continue down past the parked van,  and go underneath it | | Which side of the parked van to pass on (left) | |
| The allotments | | | Go up and past the allotments | | Whether to pass below or above the allotments (above) | |
| The museum | | | Go over to the right side of the museum | | Whether to pass below or above the museum (below) | |
| The disused monastery | | | Head upwards and then turn left at the disused  monastery | | Whether to pass below or above the monastery (above) | |
| Alpine garden | | | Go up and past the alpine garden | | Whether to pass on the left or right (right) | |
| The youth hostel | | | Go past the youth hostel | | Whether to go above or below the hostel (above) | |
| The phone box | | | Head straight down to the right side of the phone  box | | Whether to go above or below the phone box (below) | |
| The thatched mud hut | | | Go up past the thatched mud hut | | Whether to pass on the left or the right of the mud hut  (right) | |
| East lake | | | Go past the east lake to the end. | | Whether to go above or below the east lake (below) | |
| **Set B** | |  | |  | |  |
| The broken gate | | | From the start go down past the broken gate | | Whether to pass on the left or right (right) | |
| The picnic site | | | Continue down past the picnic site and go underneath it/stop underneath it | | Whether to pass on the left or right (right) | |
| The children’s play area | | | You go up and past the children’s play area | | Whether to go above or below the play area (above) | |
| The train crossing | | | Go over to the right side of the train crossing | | Whether to go above or below the train crossing  (below) | |
| The granite quarry | | | Go up and past the granite quarry | | Whether to go above or below the granite quarry  (above) | |
| The waterfall | | | Go round the waterfall | | Whether to pass the waterfall on the left or right (right) | |
| The signpost | | | Go past the signpost | | Whether to go above or below the signpost (above) | |
| The haystack | | | Head straight down to the left side of the  haystack | | Whether to go above or below the haystack (below) | |
| The steep cliffs | | | Go past the steep cliffs | | Whether to pass the cliffs on the left or right (right) | |
| The west lake | | | Go past the west lake to the end | | Whether to go above or below the lake (below) | |

Supplementary Table 2: Directional responses and corresponding key inputs for the Wizard-of-Oz Map task system.

| **Key input** | | | **Response** | |
| --- | --- | --- | --- | --- |
| ***Direct questions*** |  | | | |
| Y | | | Yes. | |
| N | | | No. | |
| IDK | | | I don’t know. |  |
| ***Direction request*** |  | | | |
| L | | | Left. | |
| LV | | | On the left. | |
| R | | | Right. | |
| RV | | | On the right. | |
| A | | | Above. | |
| AV | | | You go above it. | |
| B | | | Below. | |
| BV | | | You go below it. | |
| U | | | Upwards. | |
| UV | | | You go upwards. | |
| D | | | Downwards. | |
| DV | | | You go downwards. | |
| ***Ad-hoc responses*** | |  | | |
| NP | | | I can’t give you any past instructions, let’s just move on from here. | |
| NS | | | I’m not sure, I don’t have that information in my script. | |
| REP | | | Sorry, could you repeat that? | |
| ONE | | | I can only give you one bit of information at a time, what would you like to know first? | |
| WHICH | | | Which landmark is easiest for you to go from? | |
| PAUSE | | | Hang on, I’ll just check. | |
| Q | | | Let’s go from [the last landmark passed]. | |
| F | | | [The landmark for the current statement] | |
| G | | | [Repeat last statement] | |
| H | | | [Repeat initial instruction] | |
| I | | | [Move on to next instruction] | |

Supplementary Table 3: Counterbalancing grid of human and computer order of administration with Map versions A and B.

|  | **Human A, Computer B** | **Computer A, Human B** | **Human B, Computer A** | **Computer B, Human A** |
| --- | --- | --- | --- | --- |
| **Participants** | 1, 5, 9, 13, 17, 21 | 2, 6, 10, 14, 18, 22 | 3, 7, 11, 15, 19, 23 | 4, 8, 12, 16, 20, 24 |

Supplementary Table 4: Time to complete model comparison of linear mixed effects models by REML, with best fitting model shown in bold.

| **Model** | **Fixed effects** | **Random effects** | **Df** | **AIC** | **BIC** | **LogLik** | **Deviance** | **ChiSq** | **Chi Df** | **p (ChiSq)** |
| --- | --- | --- | --- | --- | --- | --- | --- | --- | --- | --- |
| 1 (null) | None | (1+ Condition \| Participant) | 5 | 1380.5 | 1395.3 | -685.25 | 1370.5 |  |  |  |
| 2 | Condition | (1+ Condition \| Participant) | 6 | 1381.8 | 1399.6 | -684.91 | 1369.8 | 0.68 | 1 | 0.41 |
| **3** | **Condition + Trial** | **(1+ Condition \| Participant)** | **7** | **1316.8** | **1337.6** | **-651.42** | **1302.8** | **66.97** | **1** | **2.762e-16 ***** |
| 4 | Condition * Trial | (1+ Condition \| Participant) | 8 | 1317.1 | 1340.8 | -650.54 | 1301.1 | 1.76 | 1 | 0.18 |

Supplementary Table 5: Number of turns taken model comparison of linear mixed effects models by REML, with best fitting model shown in bold.

| **Model** | **Fixed effects** | **Random effects** | **Df** | **AIC** | **BIC** | **LogLik** | **Deviance** | **ChiSq** | **Chi Df** | **p (ChiSq)** |
| --- | --- | --- | --- | --- | --- | --- | --- | --- | --- | --- |
| 1 (null) | None | (1+ Condition \| Participant) | 5 | 985.46 | 1000.31 | -487.73 | 975.46 |  |  |  |
| 2 | Condition | (1+ Condition \| Participant) | 6 | 984.28 | 1002.10 | -486.14 | 972.28 | 3.18 | 1 | 0.07 |
| **3** | **Condition + Trial** | **(1+ Condition \| Participant)** | **7** | **943.24** | **964.03** | **-464.62** | **929.24** | **43.05** | **1** | **5.346e-11 ***** |
| 4 | Condition * Trial | (1+ Condition \| Participant) | 8 | 942.87 | 966.63 | -463.44 | 926.87 | 2.36 | 1 | 0.12 |

Supplementary Table 6: Delayed recall accuracy model comparison of linear mixed effects models by REML, with best fitting model shown in bold.

| **Model** | **Fixed effects** | **Random effects** | **Df** | **AIC** | **BIC** | **LogLik** | **Deviance** | **ChiSq** | **Chi Df** | **p (ChiSq)** |
| --- | --- | --- | --- | --- | --- | --- | --- | --- | --- | --- |
| 1 (null) | None | (1+ Time \| Participant) | 5 | 937.80 | 952.65 | -463.90 | 927.80 |  |  |  |
| 2 | Condition | (1+ Time \| Participant) | 6 | 930.79 | 948.61 | -459.40 | 918.79 | 9.01 | 1 | 0.003 *** |
| **3** | **Condition + Delay** | **(1+ Time \| Participant)** | **8** | **830.02** | **853.78** | **-407.01** | **814.02** | **104.77** | **2** | **2.2e-16 ***** |
| 4 | Condition * Delay | (1+ Time \| Participant) | 10 | 833.57 | 863.27 | -406.78 | 813.57 | 0.45 | 2 | 0.80 |
